# Trachoma in 3 Amerindian Communities, Venezuelan Amazon, 2018

**DOI:** 10.3201/eid2501.181362

**Published:** 2019-01

**Authors:** Oscar Noya-Alarcón, Maríapía Bevilacqua, Alfonso J. Rodríguez-Morales

**Affiliations:** Universidad Central de Venezuela, Caracas, Venezuela (O. Noya-Alarcón);; Asociación Venezolana para la Conservación de Áreas Naturales, Caracas (M. Bevilacqua);; Universidad Tecnológica de Pereira, Pereira, Colombia (A.J. Rodríguez-Morales);; Universidad Privada Franz Tamayo, Cochabamba, Bolivia (A.J. Rodríguez-Morales)

**Keywords:** Trachoma, Chlamydia trachomatis, blindness, neglected tropical diseases, Venezuela, indigenous population, Amerindians, ocular infection, bacteria

## Abstract

Trachoma is among the most common infectious causes of blindness. During January–May 2018, a total of 4 trachoma cases were diagnosed among Amerindians of the Yanomami ethnic group in 3 communities of southern Venezuela. This country has social and environmental conditions conducive to the endemicity of this neglected tropical disease.

Trachoma, caused by the bacterium *Chlamydia trachomatis*, is the most common infectious cause of blindness. It is endemic to many of the poorest and most remote areas of Africa, Asia, Australia, the Middle East, and Latin America (*1*). Trachoma causes visual impairment in ≈2.2 million persons worldwide, of whom 1.2 million are completely blind (*2*). As of April 2018, ≈158 million persons living in districts to which trachoma is endemic are at risk (*3*). In South America, trachoma is considered endemic to Brazil (*4*) and Colombia (*5*) but not to Venezuela. We describe 4 patients in whom trachoma was diagnosed during January–May 2018 in 3 communities in the Amazon region of southern Venezuela. All were Amerindians of the Yanomami ethnic group living near rivers in extensive, well-conserved international forest frontiers.

During January–May 2018, in the integrated health care system in the Venezuela states of Amazonas and Bolivar, 4 trachoma cases were detected. Two cases occurred in the Yanomami community of Kuyuwiniña, Alto Caura River basin, Bolivar, and 1 case occurred in each of 2 communities of the upper Orinoco River basin of Amazonas (Oroshi and Rashakami) (Appendix Figure 1).

Case-patient 1 was a 38-year-old woman from Oroshi with a 5-month history of trachomatous trichiasis (TT), pain, madarosis, blepharitis, and conjunctivitis in both eyes. Case-patient 2 was a 35-year-old woman from Kuyuwiniña with a 6-month history of TT, pain, madarosis, blepharitis, and conjunctivitis in both eyes; corneal opacity in the right eye; and full blindness in the left eye (Appendix Figure 2). Case-patient 3 was a 45-year-old man from Kuyuwiniña with a 5-year history of TT, madarosis, blepharitis, conjunctivitis, and corneal opacity in both eyes. Case-patient 4 was a 22-year-old man from Rashakami with a 1-year history of TT, madarosis, blepharitis, and keratitis in both eyes; full blindness in the left eye; and decreased vision in the right eye. 

All 4 patients used natural depilatory wax to improve their trachoma. No additional information on use of traditional eye medicine or epilation was obtained. These communities have no access to potable water except rivers and live crowded in open aboriginal community households (Appendix Figure 1, panel B). These patients were treated with azithromycin (1 g single dose orally) and showed clinical improvement (less inflammation) 3 months later (*6*) without surgery.

Trachoma is a neglected tropical disease (NTD) that disproportionately affects the poorest communities (*7*). Worldwide, many indigenous peoples are at risk (*4*,*5*,*7*). The geographic origin of these cases is unknown. In remote areas of the southern Venezuelan Amazon, the population moves within the Caura River basin and in the upper Orinoco basin and to and from Brazil in the headwaters of the Auaris River and other subbasins of the Branco River in Yanomami territory. The potential introduction of infected illegal gold miners also should be considered as a source of trachoma. The remoteness of these communities often means they have limited access to healthcare, making assessment of trachoma and other diseases challenging. Thus, findings of this NTD and others is not surprising. 

Trachoma was originally reported in Venezuela in 1894; at least 17 cases were sporadically reported during 1903–1956. In 1982, six case-patients (2 female, 4 male) 30 months–22 years of age were described (*8*).

A resolution of the World Health Assembly in 1998 established political commitment for global elimination of trachoma as a public health problem. Much progress is being made toward that goal, but momentum may be insufficient to meet the 2020 target (*1*), particularly given emerging evidence of previously unknown endemic foci in places such as Venezuela and the Democratic Republic of the Congo. Population-based studies are needed to define the prevalence of trachoma in these communities of Venezuela, which border Brazil, a country in which this NTD is endemic in indigenous populations, with reported prevalences of up to 35.2% for the trachomatous follicular inflammation in children 1–9 years of age (*4*).

Water is necessary for face washing, and trachoma often occurs in communities or households without an adequate water supply. Several studies have identified a positive association between the distance to the water source and the prevalence of active trachoma (*1*). Because of improvement of socioeconomic and sanitary status (*9*), advent of new generations of antimicrobial drugs, and training of ophthalmologists and eye-care facilities, the prevalence of trachoma is decreasing (*2*). In the context of the onchocerciasis elimination program in the area, ophthalmologists and other specially trained physicians periodically attended these populations to assess visual health, including onchocercosis. In countries such as Brazil (*4*) and Colombia (*5*), trachoma appears to be a serious public health problem in indigenous settlements and should be prioritized in programs aimed at eliminating trachoma (*1*,*2*,*7*). The cases we report suggest that national and international health authorities should consider developing surveillance and undertaking research for trachoma in these areas of Venezuela (*6*,*10*).

Appendix Location of trachoma cases in 3 Amerindian communities, Amazonian Venezuela, 2018; case-patient showing the effects of trachoma.
