# Supplementary material for: Trachoma in 3 Amerindian Communities, Venezuelan Amazon, 2018
Source: Emerg Infect Dis. 2019 Jan;25(1):182–3. doi: 10.3201/eid2501.181362 (PMC6302605; doi:10.3201/eid2501.181362)
Supplement: Appendix — Location of trachoma cases in 3 Amerindian communities, Amazonian Venezuela, 2018; case-patient showing the effects of trachoma. [file 18-1362-Techapp-s1.pdf]

Article DOI: https://doi.org/10.3201/eid2501.181362

Trachoma in 3 Amerindian Communities, Venezuelan Amazon, 2018

Appendix

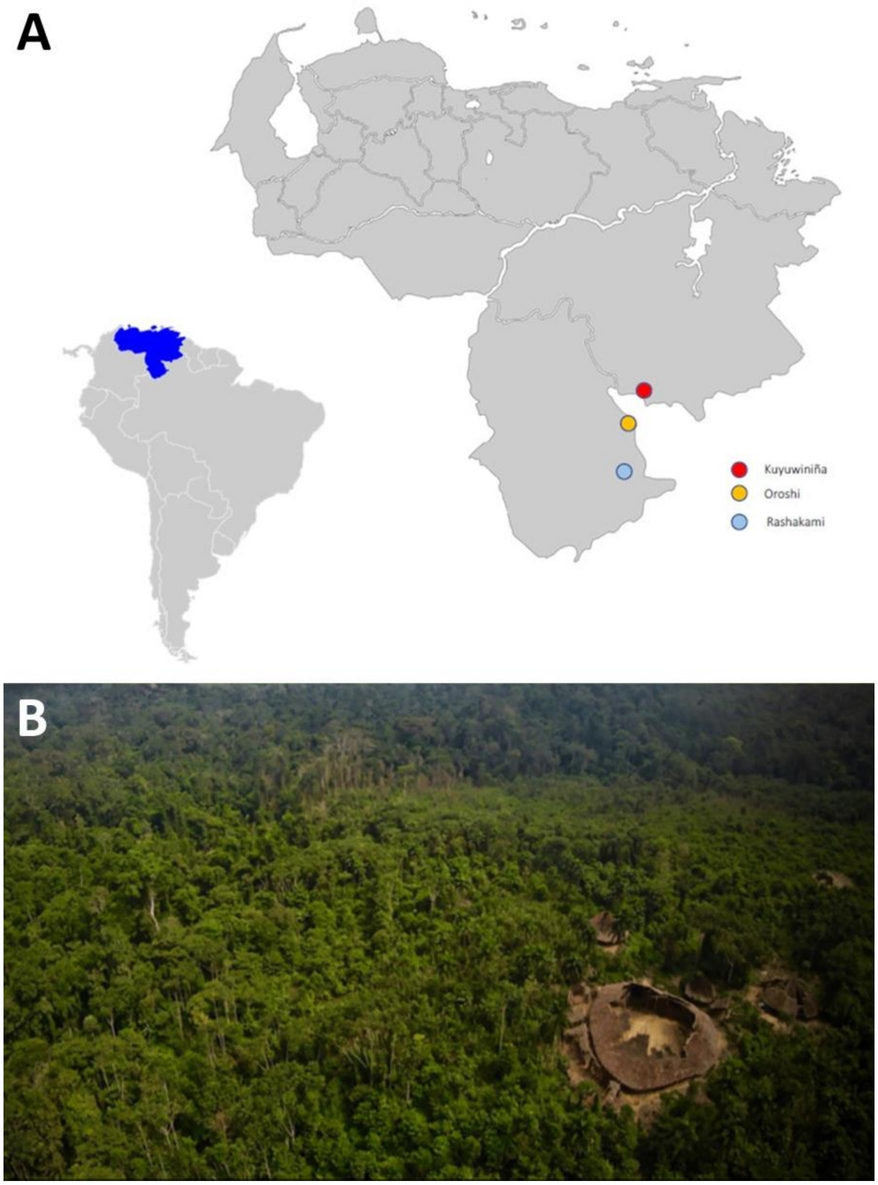

Appendix Figure 1. Study of trachoma in 3 Amerindian communities, Amazonian Venezuela, 2018. A) Location of study areas. B) Kuyuwiniña community, Alto Caura River basin, Bolivar.

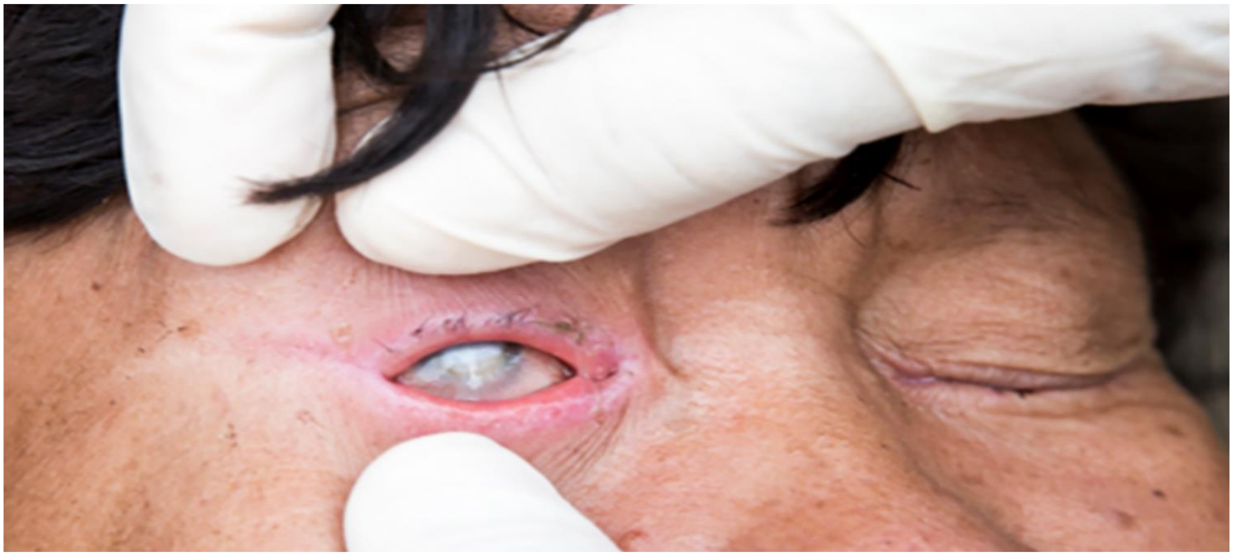

Appendix Figure 2. Case-patient 2, a 35-year-old woman, with trachomatous trichiasis, madarosis, blepharitis, and conjunctivitis in both eyes; corneal opacity in the right eye; and full blindness in the left, Community Kuyuwiniña community. Alto Caura River basin, Bolivar.
